# Correction: Shared genetics between breast cancer and predisposing diseases identifies novel breast cancer treatment candidates

**DOI:** 10.1186/s40246-024-00707-4

**Published:** 2025-01-23

**Authors:** Panagiotis N. Lalagkas, Rachel D. Melamed

**Affiliations:** Department of Biological Sciences, University of Massachusetts, Lowell, MA USA

**Human Genomics (2024) 18:124**

10.1186/s40246-024-00688-4

The original article mistakenly presented an error whereby a main text paragraph was inadvertently swapped with the Fig. 3 caption; this has since been amended.

