# Supplementary material for: Shared genetics between breast cancer and predisposing diseases identifies novel breast cancer treatment candidates
Source: Hum Genomics. 2024 Nov 14;18:124. doi: 10.1186/s40246-024-00688-4 (PMC11562851; doi:10.1186/s40246-024-00688-4)
Supplement: Supplementary file 6 — Supplementary Material 6 [file 40246_2024_688_MOESM6_ESM.pdf]

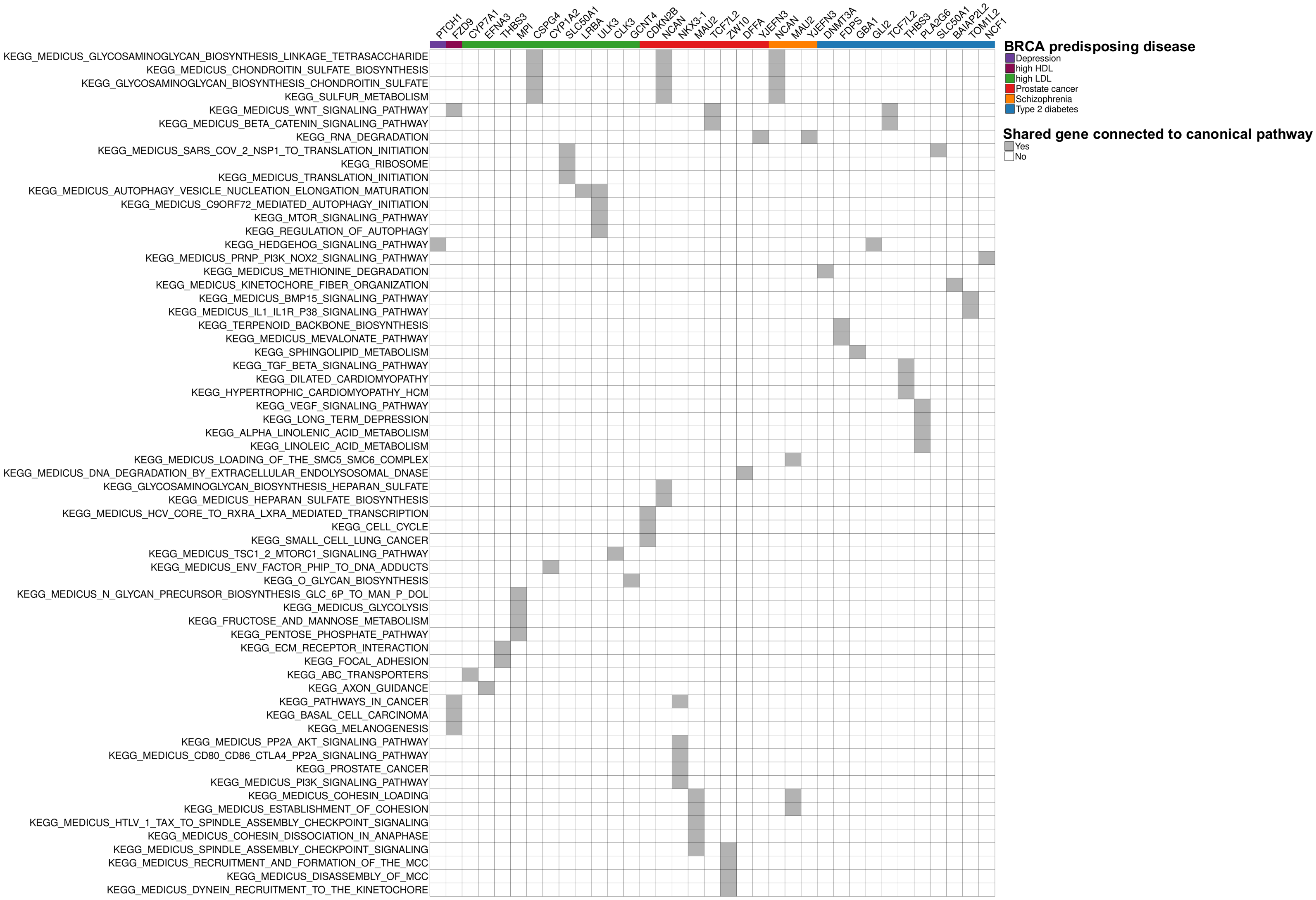

Supplementary Figure 1. KEGG canonical pathways connected to the shared genes between breast cancer and its predisposing diseases.
